# A high-quality chromosome-level genome assembly of *Ficus hirta*

**DOI:** 10.1038/s41597-024-03376-z

**Published:** 2024-05-22

**Authors:** Weicheng Huang, Yamei Ding, Songle Fan, Wanzhen Liu, Hongfeng Chen, Simon Segar, Stephen G. Compton, Hui Yu

**Affiliations:** 1 Plant Resources Conservation and Sustainable Utilization, the Chinese Academy of Sciences, Guangzhou, 510650 China; 2 State Key Laboratory of Plant Diversity and Specialty Crops, South China Botanical Garden, the Chinese Academy of Sciences, Guangzhou, 510650 China; 3 Department of Crop and Environment Sciences, Harper Adams University, Newport, Shropshire TF10 8NB UK; 4 School of Biology, University of Leeds, Leeds, LS2 9JT UK; 5 State Key Laboratory of Plant Diversity and Specialty Crops, South China Botanical Garden, Chinese Academy of Sciences, Guangzhou, Guangdong 510650 China

**Keywords:** Phylogenetics, Comparative genomics, Genome evolution

## Abstract

*Ficus* species (Moraceae) play pivotal roles in tropical and subtropical ecosystems. Thriving across diverse habitats, from rainforests to deserts, they harbor a multitude of mutualistic and antagonistic interactions with insects, nematodes, and pathogens. Despite their ecological significance, knowledge about the genomic background of *Ficus* remains limited. In this study, we report a chromosome-level reference genome of *F. hirta*, with a total size of 297.27 Mb, containing 28,625 protein-coding genes and 44.67% repeat sequences. These findings illuminate the genetic basis of *Ficus* responses to environmental challenges, offering valuable genomic resources for understanding genome size, adaptive evolution, and co-evolution with natural enemies and mutualists within the genus.

## Background & Summary

*Ficus* is a highly species rich genus of mainly pantropical woody plants with a diverse range of growth forms. Fig trees occupy a broad range of habitats^1,2^ and are among the most ecologically important plant groups in tropical forests^3,4^. The genus is characterized by its enclosed inflorescences (figs, also called syconia) that vary in size and location, but have remained unchanged in fundamental structure since the genus first appeared around 45 mya^5–7^. The evolutionary history of the genus has therefore combined extensive radiation and ecological diversification with a reproductive conservatism that is linked to their unique interaction with the trees’ only pollinators (fig wasps, Hymenoptera Agaonidae). Perhaps the most significant innovation involving fig anatomy has involved the modification of breeding systems, with some *Ficus* species monoecious, others gynodioecious (but functionally dioecious), that involves associated changes in floral anatomy^8^. *Ficus* belongs to the Eudicot family Moraceae, placed by recent phylogenies within the ‘urticalean’ clade of Rosales. Dioecy is believed to be the ancestral state within Moraceae as a whole^5^ but the ancestral breeding system in *Ficus* remains uncertain^8^. Most *Ficus* species are diploid with 2n = 26, irrespective of their phylogenetic relations within the genus^9^, but tetraploid species are known from Africa^10^. The significance of hybridization in *Ficus* diversification has been debated, but Gardner *et al*. have shown that while introgression has taken place, it has not had a major impact on evolution in the genus^7^.

In addition to pollinating fig wasps, *Ficus* also has symbiotic non-pollinating fig wasps, beetles, flies, moths, nematodes and pathogens that are likely to have a negative impact on the host. More than 300 leaf-chewing and more than 400 sap-sucking insect species were recorded from just 15 *Ficus* species from Papua New Guinea^11–14^. *Ficus* species possess diversified direct defense strategies, including physical structures and differing chemical defenses^15,16^. They are known to contain hundreds of different secondary metabolites^17,18^, but we know little of the underlying genetics.

Here, we assembled a high-quality chromosome-level genome of *F. hirta* using a combination of PacBio HiFi sequencing and Hi-C techniques and compared this with previously published genomes of four congeners. The assembled *F. hirta* genome had a combined length of 297.27 Mb, featuring a contig N50 of 19.71 Mb and achieving a complete BUSCO score of 98.50%. A substantial 282.12 Mb (94.90%) of the sequences were successfully anchored to the 13 pseudochromosomes. The genome annotation predicted 28,625 protein-coding genes. This high-quality *F. hirta* genome provides novel genomic resources for future researchers on genome and adaptive evolution within fig trees, as well as *Ficus-*natural enemy and mutualist co-evolution.

## Methods

### Sample collection and sequencing

*F. hirta* material came from a natural population growing in the South China Botanical Garden (23.18°N, 113.36°E), Guangzhou, China. Fresh young leaves of *F. hirta* were collected for genome sequencing. Organs (leaves, stems, inflorescences and roots) were collected from three individual trees to provide biological replicates of the *F. hirta* sampled for its transcriptome. All samples were immediately flash-frozen using liquid nitrogen and stored at −80 °C for subsequent nucleic acid extraction. High-quality genomic DNA was isolated from young leaves of *F. hirta* using the CTAB method^19^. The genomic DNA was then fragmented into random fragments, and short-read libraries of *F. hirta* were constructed according to Illumina’s standard protocol, and paired-end reads (150 bp) were sequenced on an Illumina NovaSeq platform. Additionally, a 15 kb HiFi library was constructed following the protocol for the PacBio Sequel2 platform, and circular consensus sequencing (CCS) was performed. A Hi-C library^20^ was also sequenced on an Illumina NovaSeq platform with paired-end reads of 150 bp. Total RNA was extracted using CTAB and RNA-seq libraries were constructed and sequenced on an Illumina NovaSeq platform with a read length of 150 bp on both sides. All Illumina sequencing data were filtered to obtain clean data using the fastp v0.23.1 software^21^ for subsequent analysis. All analyses were performed on a laboratory server with 60 TB storage and 100 threads, operating on Linux.

### Genome assembly

Before assembly, we first estimated the genome size and heterozygosity of *F. hirta* by calculating the 17-mer frequency distribution using Jellyfish v2.3.0 and GenomeScope v2.0 software^22,23^. Next, Pacbio HiFi reads were assembled into contigs using hifiasm v0.15.4 with the default parameters^24^. To obtain clean Hi-C data, we used HiC-Pro v3.1.0 to filter the raw Hi-C data^25^. After that, the clean Hi-C data were aligned to the final assembled contigs by the juicer pipeline v1.6 to obtain the interaction matrix^26^. The contigs were then ordered and anchored using 3D *de novo* assembly (3D-DNA) v180419^27^. Finally, the Hi-C contact maps of the final assembly result were reviewed manually with Juicebox v1.11.08^26^.

**Fig. 1:**
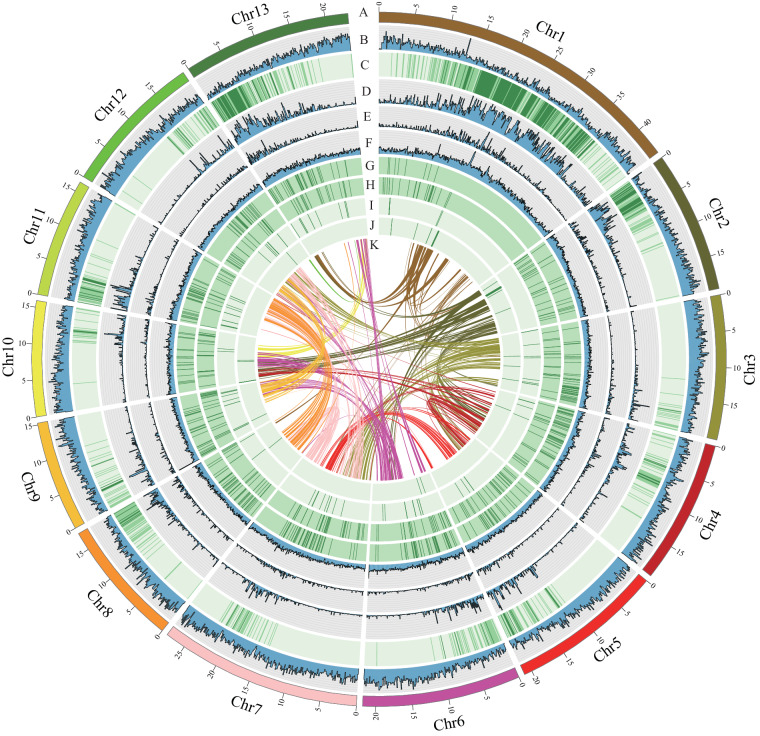
The genomic features of *Ficus hirta*. (**A**) The 13 pseudochromosomes; (**B**) gene density; (**C**) histogram of GC content; (**D**–**F**) the density of total repeat sequences, Gypsy LTR-REs, and Copia LTR-REs; (**G**–**J**) tRNA, snRNA, miRNA, and rRNA density; (**K**) intragenomic collinearity. (**B**–**J**) were drawn in 100 kb overlapping sliding windows.

The genome of *F. hirta* was estimated to be 283.52 Mb in size, with a heterozygosity of 1.26% (Figure S1). We performed *de novo* assembly of the *F. hirta* genome at the chromosome-level based on PacBio reads generated in CCS mode (HiFi reads), with 31.76 Gb (106-fold coverage), 65-fold coverage of clean Illumina short reads amounting to 19.49 Gb, and 124-fold coverage of high-throughput chromatin conformation capture (Hi-C) data amounting to 37.05 Gb (Table S1). The assembled genome size was 297.27 Mb, with 282.12 Mb anchored onto 13 pseudochromosomes (anchor rate: 94.90%) (Fig. 1A; Figure S2; Table 1). The contig N50 was 19.71 Mb, which has higher integrity and continuity (contigs N50: 0.18 to 2.29 Mb) (Table S2), compared to *F. carica* (8.23 Mb)^28^, *F. microcarpa* (1.77 Mb)^29^, *F. hispida* (2.16 Mb)^29^, and *F. religiosa* (5.53 Mb)^30^.

**Table 1:** Statistics for published *Ficus* genomes.

Genome assembly	
the genome scaffolds number	26
the genome contigs number	55
the longest length (bp)	44,007,737
the shortest (bp)	50,000
Genome size (bp)	297,279,994
the rate of GC (%)	34.88
the scaffold N50 (bp)	19,920,111
the scaffold L50	6
the contig N50 (bp)	19,716,268
the contig L50	7
Anchor rate (%)	94.9
BUSCOs (%)	98.5
LAI	19.98
Genome annotation	
No. of protein-coding genes	28,625
Average gene length (bp)	3,419
Percentage of repetitive sequences (%)	39.86

### Genome annotation

For repeated elements identification and masking, we used homology-based and *de novo* approaches to identification. Briefly, a *de novo* repeat library was constructed using RepeatModeler v2.0.2^31^. Then the obtained library was combined with the Repbase database v21.12^32^ to identify repetitive sequences in the *F. hirta* genome using RepeatMasker v4.1.2^33^. For noncoding RNA prediction, the tRNA genes were predicted using tRNAscan-SE v2.0.6^34^. Others, including miRNA, rRNA and snRNA genes, were detected by comparison with the Rfam database using CMsearch v1.1.3 with the default parameters^35,36^. Protein-coding gene annotation was conducted using homology-based, transcriptome-based, and *ab initio* prediction methods. First, we used homologies from 11 different species (Table S3) as protein-based evidence for predicting gene sets using GeneWise v2.4.1^37^. Transcriptome data, including leaf, stem, inflorescence, and root RNA-seq reads were mapped using HISAT2 v2.1.0^38^. *Ab initio* prediction using packages AUGUSTUS v3.4.0^39^, trained by the transcriptome data. To generate a comprehensive protein-coding gene set, we used the GETA pipeline (https://github.com/chenlianfu/geta) to integrate annotations from all homology-based, transcriptome-based, and *ab initio* predictions. To functionally annotate the predicted gene models, we searched several different databases, including the NCBI nr^40^, Swiss-Port^41^, KOG^42^, eggNOG^43^, Pfam^44^, GO^45^, and KEGG^46^.

In total, 28,625 protein-coding genes were predicted using a combination of de novo homolog-based searches and RNA-seq data, of which 92.39% could be functionally annotated (Fig. 1B,C; Table 1; Table S4). The predicted proteome contained 98.50% complete and 0.80% fragmented BUSCO genes (Table S5). A total of 132.79 Mb repeat elements were identified, which accounted for 44.67% of the *F. hirta* genome (Fig. 1D; Table 2). The most abundant repetitive elements were LTR retrotransposon (LTR-RE) elements (59.31 Mb; LTR-RE/Copia: 13.59 Mb; LTR-RE/Gypsy: 41.60 Mb), followed by DNA transposons (11.58 Mb), with an additional 46.13 Mb of unclassified repetitive sequences (Fig. 1E,F; Table 2). Furthermore, our analysis revealed the presence of 9,830 noncoding RNAs, which included 133 miRNAs, 574 transfer RNAs (tRNA), 8,717 ribosomal RNAs (rRNA), and 406 small nuclear RNAs (snRNA) (Fig. 1G–J; Table S6).

**Table 2:** Statistics of repeat sequences in *Ficus hirta* genome.

Type	number of elements	length occupied of sequence (bp)	Percentage (%)
Class I: Retroelements	67,885	60,768,272	20.44
SINEs	82	10,262	0.00
LINEs	3,959	1,442,540	0.49
L2/CR1/Rex	720	272,805	0.09
R2/R4/NeSL	149	28,863	0.01
RTE/Bov-B	660	266,005	0.09
L1/CIN4	2,430	874,867	0.29
LTR elements	63,844	59,315,470	19.95
Ty1/Copia	24,901	13,596,523	4.57
Gypsy/DIRS1	32,826	41,608,009	14.00
Class II: DNA transposons	26,491	11,586,496	3.90
hobo-Activator	6,858	2,129,275	0.72
Tc1-IS630-Pogo	101	27,592	0.01
Tourist/Harbinger	3,813	1,251,457	0.42
Rolling-circles	1,722	1,811,588	0.61
Unclassified	206,166	46,130,154	15.52
Small RNA	5,523	1,584,467	0.53
Satellites	482	218,225	0.07
Simple repeats	246,733	8,768,605	2.95
Low complexity	37,986	1,935,042	0.65
Total	—	132,792,587	44.67

## Data Records

The National Genomics Data Center (NGDC) database BioProject accession number for the sequence reported in this paper is PRJCA019243. The raw sequencing data for HiFi, Hi-C, and RNA-seq were submitted to NGDC GSA with accession numbers CRR857341-CRR857356^47^. The chromosomal-level genome assembly file was deposited in the NCBI GenBank with accession number GCA_038430175.1^48^. Moreover, the gene structure annotation, gene function annotation and TE annatition files have been deposited at the Figshare^49^ database.

## Technical Validation

To assess genome assembly quality, the Illumina genomic and RNA-seq reads were mapped to the genome using BWA v0.7.17^50^ and HISAT2 v2.1.0^38^, respectively. To evaluate the completeness and accuracy of the genome, we used the LTR assembly index (LAI)^51^ and BUSCO v4.1.2^52^ evaluation with the embryophyta_odb10 database to examine. Finally, the mapping rates of Illumina and HiFi reads to the genome were 98.52% and 99.13%, respectively (Table S7). The LAI had a score of 19.98 (Table 1), which is similar to the scores for *Oryza sativa* and *Arabidopsis thaliana*^51^. Benchmarking Universal Single-Copy Orthologs (BUSCO) analyses showed the assembled genome contained 1,590 (98.50% of 1,614) complete sets of the core orthologous genes in the Embryophyta_odb10 database, which is higher than that of the seven previously reported *Ficus* genomes (89.7%–96.4%) (Table S5). All these values suggest a high quality of *F. hirta* genome sequence.

### Supplementary information

Supplementary Figures

Supplementary Tables

## Data Availability

No custom code was used for this study. All sofware and pipelines were executed according to the manual and protocols of the published bioinformatics tools. The version and code/parameters of sofware have been detailed described in Methods.
