# Supplementary material for: A high-quality chromosome-level genome assembly of Ficus hirta
Source: Sci Data. 2024 May 22;11:526. doi: 10.1038/s41597-024-03376-z (PMC11111794; doi:10.1038/s41597-024-03376-z)
Supplement: Supplementary file 1 — Supplementary Figures [file 41597_2024_3376_MOESM1_ESM.docx]

## Supplementary Figures


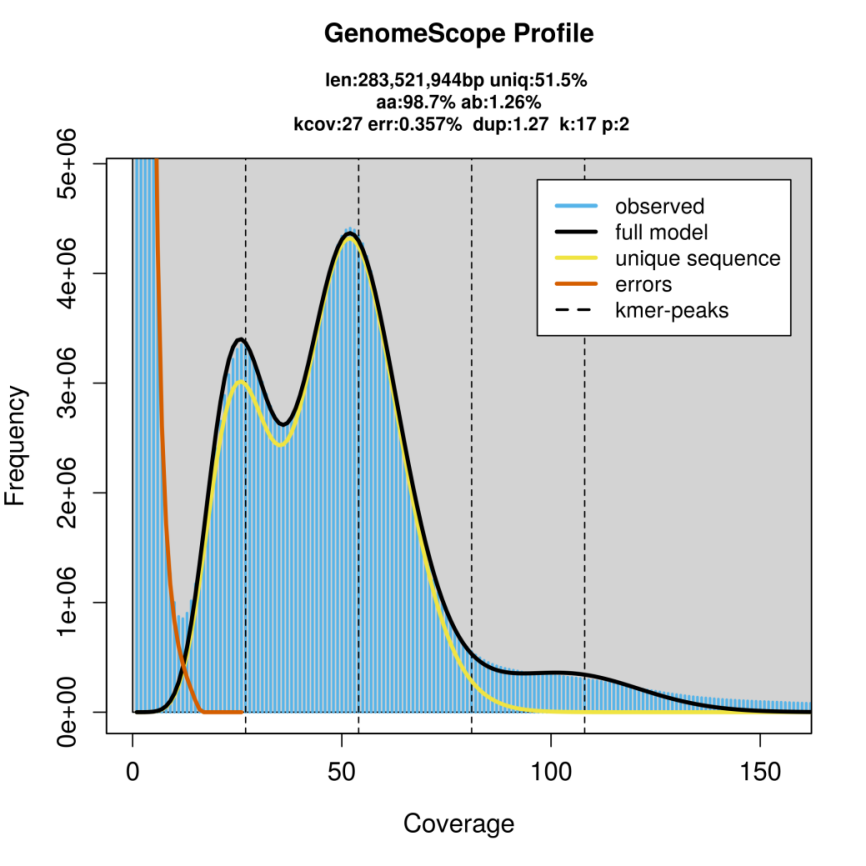


**Figure S1 |** K-mer estimation of *F. hirta* genome. The abbreviation for all species as following: len: genome length/size; uniq: unique percent (not repetitive); aa: homozygous rate; ab: heterozygous rate; het: heterozygosity; kcov: kmer coverage; err: error rate of reads; dup: duplications rate of reads; k: kmer; p: ploidy.


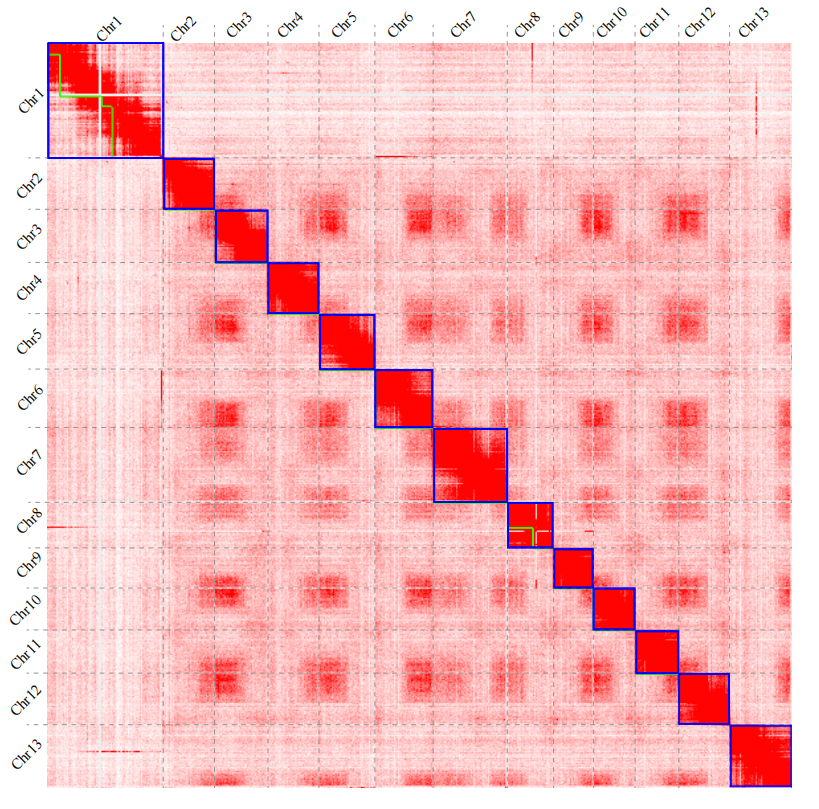


**Figure S2 |** Genome-wide chromatin interactions of *F. hirta* based on Hi-C data. Heatmaps of chromatin interactions anchored the contigs onto 13 pseudochromosomes.
